# Low Nitrogen Input Mitigates Quantitative but Not Qualitative Reconfiguration of Leaf Primary Metabolism in *Brassica napus* L. Subjected to Drought and Rehydration

**DOI:** 10.3390/plants13070969

**Published:** 2024-03-27

**Authors:** Benjamin Albert, Younès Dellero, Laurent Leport, Mathieu Aubert, Alain Bouchereau, Françoise Le Cahérec

**Affiliations:** 1 Institute for Genetics, Environment and Plant Protection (IGEPP), National Research Institute for Agriculture, Food and Environment (INRAE), Institut Agro Rennes-Angers, Université Rennes, 35650 Le Rheu, France; 2 Metabolic Profiling and Metabolomic Platform (P2M2), MetaboHUB-Grand-Ouest, 31400 Toulouse, France

**Keywords:** nitrogen deficiency, water status, sink-source relationships, nitrogen and carbon metabolisms, amino acids, sugars, organic acids

## Abstract

In the context of climate change and the reduction of mineral nitrogen (N) inputs applied to the field, winter oilseed rape (WOSR) will have to cope with low-N conditions combined with water limitation periods. Since these stresses can significantly reduce seed yield and seed quality, maintaining WOSR productivity under a wide range of growth conditions represents a major goal for crop improvement. N metabolism plays a pivotal role during the metabolic acclimation to drought in *Brassica* species by supporting the accumulation of osmoprotective compounds and the source-to-sink remobilization of nutrients. Thus, N deficiency could have detrimental effects on the acclimation of WOSR to drought. Here, we took advantage of a previously established experiment to evaluate the metabolic acclimation of WOSR during 14 days of drought, followed by 8 days of rehydration under high- or low-N fertilization regimes. For this purpose, we selected three leaf ranks exhibiting contrasted sink/source status to perform absolute quantification of plant central metabolites. Besides the well-described accumulation of proline, we observed contrasted accumulations of some “respiratory” amino acids (branched-chain amino acids, lysineand tyrosine) in response to drought under high- and low-N conditions. Drought also induced an increase in sucrose content in sink leaves combined with a decrease in source leaves. N deficiency strongly decreased the levels of major amino acids and subsequently the metabolic response to drought. The drought-rehydration sequence identified proline, phenylalanine, and tryptophan as valuable metabolic indicators of WOSR water status for sink leaves. The results were discussed with respect to the metabolic origin of sucrose and some amino acids in sink leaves and the impact of drought on source-to-sink remobilization processes depending on N nutrition status. Overall, this study identified major metabolic signatures reflecting a similar response of oilseed rape to drought under low- and high-N conditions.

## 1. Introduction

Winter oilseed rape (WOSR) is a major oleaginous crop in Europe with important applications for human and animal nutrition and biofuel production. WOSR has high nitrogen (N) uptake efficiency but requires an important supply of mineral N compared to other oleaginous crops to achieve its seed yield [1]. This is partly explained by a low Nuse efficiency, leading to nearly 50% of the N initially absorbed being lost or not valued during source-to-sink remobilization processes, notably through leaf abscission [2]. In the context of the reduction of N inputs applied to the field to ensure a more sustainable crop production in Europe, the optimization of N management by oilseed rape represents an important challenge to address for future crop improvement [1]. Along the crop cycle of WOSR, leaf abscission occurs during leaf senescence in the autumnal vegetative stage and during the post-winter transition from the vegetative to the reproductive stage [3]. During these steps, N is remobilized from older (source) to younger (sink) leaves and subsequently to the reproductive organs through the activation of senescence-dependent catabolic processes subordinate to the recycling, transport and reallocation of nutrients according to the sink/source balance [4,5]. Hence, source-to-sink leaf N remobilization plays a critical role in the establishment of WOSR photosynthetic biomass. Under different field environments, this autumnal plant biomass is often strongly correlated to the final seed yield [6,7]. For these reasons, maintaining an important WOSR biomass during the vegetative phase represents a crucial objective for plant breeding and crop improvement. Climate change will increase the intensity and duration of abiotic stress episodes such as heat and drought waves [8]. These events are known to cause severe penalties to plant growth, development and, subsequently, seed yield [9]. Thus, maintaining seed production (quantity and quality) under a wide range of growth conditions represents a major goal for crop improvement. For this purpose, the metabolic control of nutrient acquisition and remobilization processes under combined stresses could be of great interest.

In the context of climate change and the reduction of mineral nitrogen (N) inputs applied to the field, WOSR production systems could be confronted sequentially with low-N conditions and water shortage during the autumnal vegetative stage. Plant adaptation to N deficiency or drought stress generally induces a decrease in photosynthesis and cell growth combined with an activation of nutrient recycling processes in order to maintain plant biomass [10,11]. Under such conditions, photosynthetic deficiency is due to either a reduction of the content of fundamental N compounds (RuBiSCO, chlorophylls) or to an abscisic acid-dependent stomatal closure to maintain leaf water content [12,13]. In both cases, the functioning of the central metabolic pathway will be reshaped to support nutrient-recycling processes from source-to-sink leaves [14]. These processes start with the degradation of chloroplast components, producing proteins and lipids that will be rerouted toward transport processes or catabolic pathways, while mitochondria will remain metabolically active until the late stages of senescence [15,16,17]. Among plant amino acids, branched-chain amino acids (BCAA), lysine, tyrosine and proline are preferentially degraded to provide carbon skeletons and reducing power for mitochondrial respiration (tricarboxylic acid (TCA) cycle, energy production) [18]. During the first steps of amino acid catabolism, N groups are recovered by transamination toward glutamate/glutamine/asparagine for subsequent transport through the phloem [19]. Interestingly, amino acid catabolism is strongly activated by carbon-starving conditions, such as dark-induced senescence and drought [20,21,22]. A recent study confirmed the importance of BCAA catabolism for drought tolerance in rice [23]. Given the strong dependence of TCA cycle activity with amino acid catabolism in source leaves, both N deficiency and drought could modulate TCA cycle functioning and subsequently N remobilization. Therefore, a combination of N deficiency and drought could lead to additional negative effects on these two processes.

Nevertheless, the metabolic adaptation of plants to drought displays specific features compared to their adaptation to N deficiency [24]. Notably, drought-stressed plants can accumulate osmoprotective compounds (osmolytes) such as polyols, mono- and polysaccharides and amino acids [25]. In *Brassica* species, the two osmolytes sucrose and proline can significantly contribute to the osmotic adjustment of leaves, the scavenging of hydroxy radicals, and the protection/stabilization of proteins and membranes during drought [25]. Although it plays a positive role during acclimation to drought, proline accumulation can be relatively high in crops and could interfere with N recycling, especially under low-N conditions [26,27]. Proline has also an important role during plant rehydration: its degradation in mitochondria produces reducing power and a carbon skeleton ready to use for energetic production [28]. Besides this, plant response to drought stress can also induce important modulations of phytohormone, flavonol and anthocyanin contents [29,30]. The *de novo* biosynthesis of these specialized metabolites will require aromatic and indolic amino acids such as phenylalanine, tyrosine and tryptophan [31]. Thus, amino acid metabolism plays a preponderant role in the metabolic acclimation of plants to drought and other stress conditions [22,32]. Indeed, N deficiency seems to decrease plant sensitivity to drought while maintaining the regulation of amino acid metabolism in different plants [33,34,35,36]. Thus, the modulation of amino acid metabolism through low-N conditions could significantly modify the metabolic acclimation of WOSR to drought.

In this context, it is crucial to understand to what extent the metabolic regulations operating during N deficiency can impact the response of WOSR to drought, including for glutamate-derived BCAA and aromatic amino acids. Given the importance of source-to-sink N remobilization for WOSR, a detailed analysis of sink and source leaves will be necessary to identify metabolic markers associated with combined drought and N deficiency. To date, recent studies only defined leaf WOSR metabolite profiles during a single stress and the sink/source status of the leaves was scarcely considered [37,38,39]. Hence, to which extent low-N conditions could modulate the metabolic acclimation of WOSR to drought and the associated source-to-sink relationships remain to be defined. For this purpose, the identification of metabolic signatures during single and combined stresses (N deficiency, drought) in the sink and source leaves could significantly contribute to a better understanding of oilseed rape metabolic acclimation processes to these two abiotic stresses. The objectives of this study were to: (i) identify the qualitative and quantitative modulations of leaf central metabolism to N deficiency, drought and combined stresses; (ii) evaluate the influence of leaf sink/source status on these responses; and (iii) define relevant metabolic indicators of the leaf water status by analyzing the plasticity of these metabolic adjustments during a rehydration phase. Here, we took advantage of a previously established experiment combining N deficiency and drought/rehydration sequences during vegetative growth to investigate the acclimation process of WOSR leaf primary metabolism in response to combined stresses in sink and source leaves [40]. For this purpose, we selected three WOSR leaves reflecting the sink/source gradient from the previously established experiment. Then, metabolic analyses were carried out in order to evaluate the metabolic reconfigurations associated with WOSR response to N deficiency, drought and combined stresses. A rehydration phase was also applied to evaluate the plasticity of drought-induced metabolic changes.

## 2. Results

**Figure 1:**
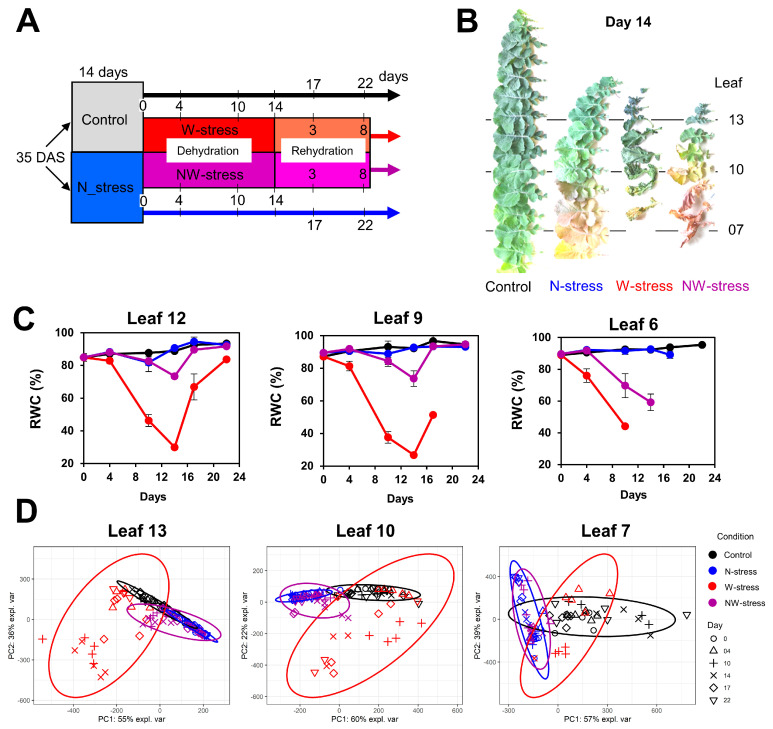
**Experimental design and leaf selection.** (**A**) Experimental setup. (**B**) Selection of leaves 13, 10 and 7 according to their sink-source status on day 14. (**C**) Validation of drought impact on leaf relative water content (RWC) during the experiment (adapted from [40]; missing points due to leaf abscission). (**D**) Principal component analysis of metabolite profiling data of leaves 13, 10 and 7 in oilseed rape plants (control, N-, W- or NW- stress) during the 22 days of experiment. After 3 weeks, plants were submitted or not to N deficiency for 2 weeks (control and N-stress conditions), followed or not by 3 weeks of drought/rehydration (W-stress and NW-stress conditions). Metabolic analyses were carried out with 3–5 biological replicates per condition, time point and leaf. The ellipses represent 95% confidence intervals for group separation by PCA. DAS = day after sowing.

In the experimental setup previously published [40], plants received high- or low-N inputs for 14 days (control and N-stress respectively). Then, half of the control and N-stress plants were subjected to a drought/rehydration sequence (W-stress, NW-stress) while the other plants remained in the same conditions (control or N-stress) (Figure 1A). Leaves 13, 10 and 7 were selected for metabolic analyses due to their contrasted sink/source physiological status in both control and stressed conditions. The young leaf 13 (sink tissue) always remained green on day 14, independently of the experimental conditions, while leaf 10 tended to have a source status (Figure 1B). Leaf 7 was an old leaf that experienced moderate-to-intensive senescence depending on the stress condition (yellowing curled leaves, leaf abscission after 14 days of drought). Drought reduced leaf relative water content (RWC) in W-stress and NW-stress conditions, but the subsequent rehydration step successfully restored leaf full hydration (Figure 1C). However, leaf ranks from NW-stress plants had lower variations of RWC compared to the leaf ranks of W-stress plants due to a lower sensitivity to drought [40]. Interestingly, a gradual variation of RWC was observed between young and mature/old leaves in W-stress and NW-stress plants. To investigate the impact of N and water availability on sink and source metabolisms of WOSR leaves, we performed a targeted absolute quantification for 52 leaf primary metabolites using UPLC-DAD and GC-FID (Figures S1 and S2). A total of 36 metabolites was successfully detected in most of the samples analyzed and comprised 25 amino acids, 4 major soluble carbohydrates, 1 polyol and 6 organic acids. A global principal component analysis (PCA) identified two major axes (PC1 and PC2) explaining 74% of the total variance but failed to distinguish the combined contribution of leaf stages and kinetic points to the separation of growth conditions (control, N-, W- and NW-stress) (Figure S3). Thus, the analysis was focused on sink and source metabolisms using separate PCAs targeting each selected leaf (13, 10 and 7) (Figure 1D). The two major PCA axes explained 82 to 96% of the total variance for each leaf and identified an important reconfiguration of central metabolism in the sink and mature leaves (13 and 10) in the W-stress condition during the drought/rehydration phases. Conversely, N- and NW-stress conditions only moderately differed from the control condition with relatively reduced metabolic variations during the kinetic period. The metabolic response of the source leaf 7 in the W-stress condition was less pronounced compared to the other leaves, although there was an important effect of the kinetic on the control condition. Subsequent analyses were based on comparisons of different conditions for each leaf in order to accurately evaluate the effect of N deficiency, drought stress, combined stresses and rehydration.

### 2.1. N Deficiency Differentially Impacts Amino Acid and Sugar Metabolism According to the Leaf Sink/Source Status

**Figure 2:**
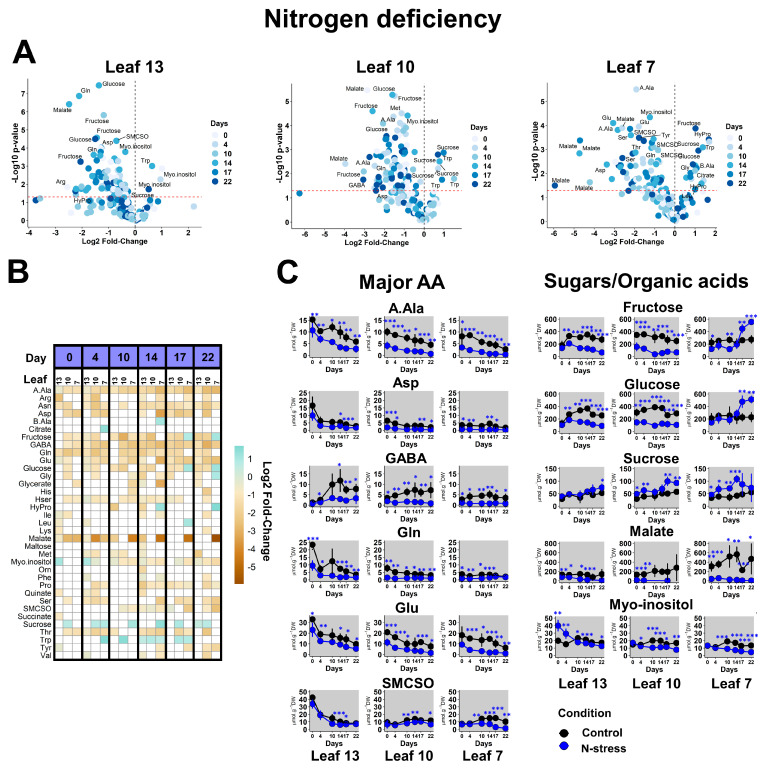
**Metabolic response of sink and sources leaves of WOSR to N deficiency.** Analyses were performed for each leaf by comparison of the metabolite contents in N-stress versus control conditions at each kinetic point. (**A**) Volcano plots. The red line indicates the confidence limit (*p*-value < 0.05). Metabolite names are indicated for the most contrasting and significant fold-changes. (**B**) Heatmap for significant fold-changes. White boxes corresponded to non-significant fold-changes. (**C**) Absolute quantifications for selected contrasted metabolites during the 22 days of the experiment. Values represent the mean ± SD for 3–5 biological replicates. Statistical differences between control and N-stress conditions at each kinetic point are denoted with blue stars according to a Student’s *t*-test (*, *p*-value < 0.05; **, *p*-value < 0.01; ***, *p*-value < 0.001). The complete dataset is available in Table S1.

Here, we used volcano plots for each leaf to identify the trend of metabolic variations between two conditions at the different time points considered (either positive (right side) or negative (left side)) and their level of statistical significance (low *p*-values for the comparisons (top side)). Overall, there was a significant reduction of metabolite content in all leaves in response to N deficiency (Figure 2A). Malate was particularly affected, with Log2 fold-changes reaching up to −6 in leaf 7 (a reduction by a factor of 64). Soluble sugars (fructose, glucose, sucrose), myo-inositol and some major amino acids (Ala, Gln, Glu, SMCSO (S-methyl-cysteine sulfoxide)) also showed an important decrease compared to other metabolites (Figure 2A,B). This metabolic pattern was already noticeable on day 0, i.e. 2 weeks after reducing the availability of N source, and persisted through the kinetic point regardless of the leaf status (Figure 2C and Figure S4). Overall, the depletions observed for the major amino acids (accounting for up to 50% of total soluble amino acid content in each leaf) reflected the depletion of total amino acid content under N-stress conditions (Figure S4B). Nevertheless, the total amino acid content in all leaves was progressively reduced in the control condition during the 22-day kinetic period, perhaps reflecting developmental leaf aging (Figure S4A and Figure 2C). Indeed, this decrease was more pronounced in leaf 13 (sink status), as reflected by Ala, Glu, Gln and Asp profiles. Regarding minor leaf amino acids, branched (Ile, Leu, Val) and aromatic (Phe, Tyr, Trp) amino acids were weakly impacted by N deficiency in both sink and source leaves (Figure 2B). The most striking differences between sink and source leaves concerned fructose and glucose, two sugars accounting for an important part of the leaf carbon pool (nearly 400 µmol·g^−1^ DW for each). The level of both metabolites was very low during N deficiency in leaves 13 and 10 but was strongly increased in leaf 7 after 17 days (Figure 2C). However, malate, another important carbon pool in WOSR, did not follow the same trend and always remained at low levels in leaf 7. Conversely, myo-inositol was found at higher levels on day 0 and day 4 in the N-deficient leaf 13 compared to the control condition. Thus, myo-inositol may be an early indicator of N deficiency in WOSR young leaves. Finally, the level of SMCSO, an important non-proteogenic amino acid in *Brassica* crops, was essentially decreased by N deficiency in leaf 7 and questioned the source-to-sink transport of SMCSO. To summarize, N deficiency induced a reduction of major amino acid contents at very low levels in all leaves and did not especially impact BCAA and aromatic amino acids. However, sugars accumulated in leaf 7 after a long period of N privation.

### 2.2. WOSR Metabolic Response to Drought Depends on Its N Status

**Figure 3:**
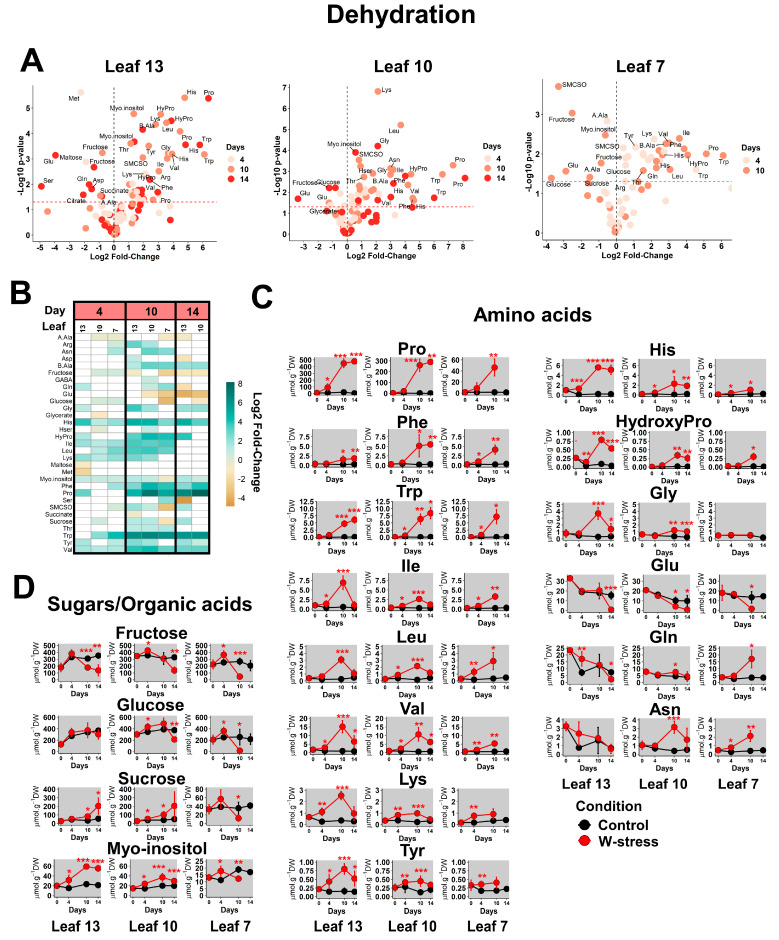
**Metabolic response of sink and sources leaves of WOSR to drought.** Analyses were performed for each leaf by comparison of the metabolite contents in W-stress versus control conditions at each kinetic point. (**A**) Volcano plots. The red line indicates the confidence limit (*p*-value < 0.05). Metabolite names are indicated for the most contrasting and significant fold-changes. (**B**) Heatmap for significant fold-changes. White boxes corresponded to non-significant fold-changes. (**C**,**D**) Absolute quantifications for selected contrasted amino acids and sugars-organic acids. Values represent the mean ± SD for 3–5 biological replicates. Statistical differences between control and W-stress conditions at each kinetic point are denoted with red stars according to a Student’s *t*-test (*, *p*-value < 0.05; **, *p*-value < 0.01; ***, *p*-value < 0.001). The complete dataset is available in Table S1.

Using volcano plots, similar global patterns for the three leaves were observed under drought (an important and significant accumulation of many amino acids), while some sugars and other amino acids were depleted (Figure 3A). Unfortunately, leaf 7 was not analyzed on day 14 due to stress-induced premature leaf abscission (Figure 1B). Nevertheless, the most significant fold-changes were observed on day 10 with a transient accumulation in the three leaves (Figure 3B,C). These results suggested a major kinetic effect rather than a sink/source-dependent regulation (Figure 1D and Figure S3). Overall, drought induced a major accumulation of Pro maximized on day 10 and day 14 but its absolute content was highly dependent on the sink/source status of the leaves (low levels in leaves 7 and 10 compared to leaf 13) (Figure 3C). The two aromatic amino acids Phe and Trp also showed an overall accumulation on days 10 and 14 compared to day 0 and day 4. Conversely, BCAAs were transitory and accumulated between day 4 and day 10 in the three leaves. Two other amino acids, Lys and Tyr, are known to be catabolized during dark-induced senescence, and other minor amino acids (His, Gly and HydroxyPro) also showed this transitory accumulation pattern, but this was essentially in leaf 13. Regarding amino acids directly involved in N assimilation and N remobilization, no major differences were observed in the youngest leaves compared to control conditions. There was essentially a significant accumulation of Gln and Asn on day 10 in leaf 7, while Glu levels were reduced at the same time. This Gln/Glu behavior is often associated with intensive source-to-sink nutrient remobilization [19]. Concerning the leaf carbon pool, the level of some major metabolites acting as carbon storage sinks in the vacuole was partially modulated by drought (glucose, fructose, citrate, malate) [41]. While malate contents were poorly modified by water stress, fructose levels were drastically reduced in the three leaves on days 10–14 by drought (Figure 3B,D). Glucose also followed a similar trend but only in the oldest leaves. Since fructose and glucose can represent an important carbon pool at the leaf level (600–800 µmol·g^−1^ DW in the control condition on day 4), such results may reflect an induction of carbon starvation by drought in all the leaves. However, the significant accumulation of sucrose and myo-inositol in leaves 13 and 10 seemed to maintain the total carbon pool of sugars and organic acids to values observed in the control condition (Figure 3D and Figure S5). Thus, carbon starvation could start on day 10 but only in the senescent leaf (250 µmol·g^−1^ DW of major sugars and organic acids for the drought condition versus 1 100 µmol·g^−1^ DW for the control condition) (Figure S5).

**Figure 4:**
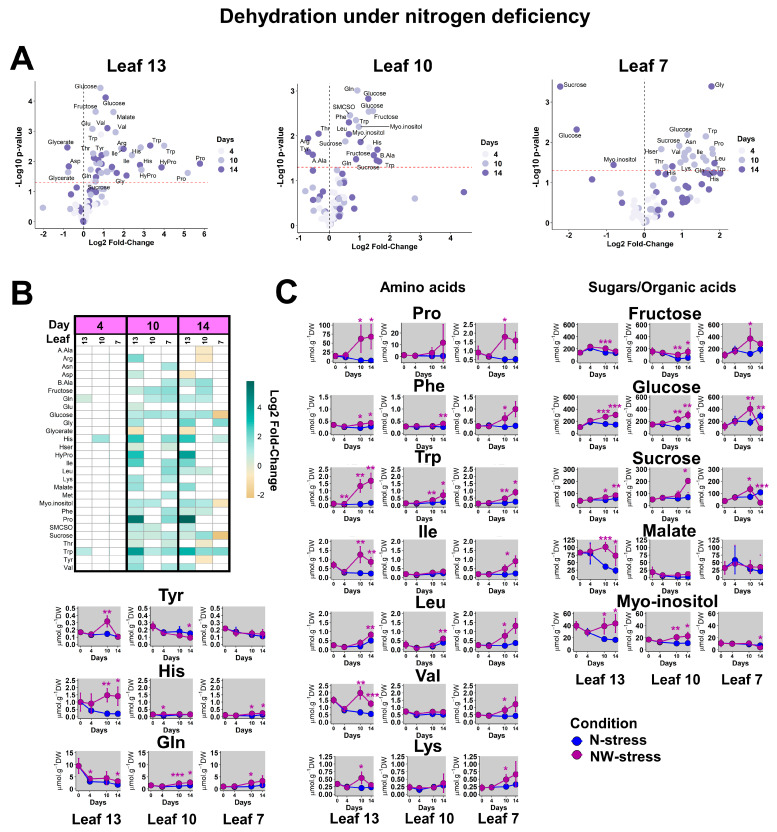
**Metabolic response of sink and sources leaves of WOSR to drought under N deficiency.** Analyses were performed for each leaf by comparison of the metabolite contents in NW-stress versus N-stress conditions at each kinetic point. (**A**) Volcano plots. The red line indicates the confidence limit (*p*-value < 0.05). Metabolite names are indicated for the most contrasting and significant fold-changes. (**B**) Heatmap for significant fold-changes. White boxes corresponded to non-significant fold-changes. (**C**) Absolute quantifications for selected contrasted metabolites. Values represent the mean ± SD for 3–5 biological replicates. Statistical differences between N-stress and NW-stress conditions at each kinetic point are denoted with purple stars according to a Student’s *t*-test (*, *p*-value < 0.05; **, *p*-value < 0.01; ***, *p*-value < 0.001). The complete dataset is available in Table S1.

To assess the impact of N deficiency on the metabolic response of oilseed rape to water stress (NW-stress), the analysis was carried out with respect to the N-stress condition to eliminate the effect of N deficiency alone. As observed for the drought condition (W-stress with high-N conditions), many metabolites were accumulated for the NW-stress condition (Figure 4A). However, the maximum values for log2 fold-changes were around 2 for leaves 10 and 7 under NW-stress compared to 6–8 under W-stress (Figure 3A and Figure 4A). Thus, the quantitative response of WOSR metabolism to drought was significantly reduced in the N-deficient old leaves. Most of the significant fold-changes occurred after 10 days of drought and persisted until the end of the water deficit for the youngest leaves (Figure 4B). Thus, the metabolic response of WOSR under the NW-stress condition was subjected to both kinetic and sink/source effects. Leaf 7 of NW-stressed plants was not subjected to stress-induced leaf abscission, perhaps suggesting an attenuation of drought perception in N-deficient plants. Indeed, the accumulations of Pro, Phe and Trp were visible on day 10 and day 14 but remained relatively lower compared to what was observed with the W-stress condition (Figure 3C and Figure 4C). BCAA, Lys and Tyr were transiently accumulated on day 10 in leaf 13 while they remained accumulated in leaf 7 on days 4, 10 and 14, except Tyr. Interestingly, Gln was significantly accumulated in leaves 10 and 7 (source status), perhaps reflecting an intensive source-to-sink nutrient remobilization. Regarding carbon metabolism, glucose, sucrose and myo-inositol were significantly accumulated in the leaves from day 10 but were depleted in leaf 7 on day 14. Overall, N deficiency clearly moderated the quantitative response of WOSR metabolism to drought. However, some key metabolic markers of WOSR response to drought, such as Pro, Phe, or Trp accumulations, were maintained in N-deficient plants. Two metabolic patterns were specifically attributed to NW-stressed plants: a continuous accumulation of sugars in sink leaves and a continuous accumulation of “respiratory” amino acids in source leaves.

### 2.3. Identification of WOSR Metabolic Markers of the Water Status under High- and Low- N Inputs

**Figure 5:**
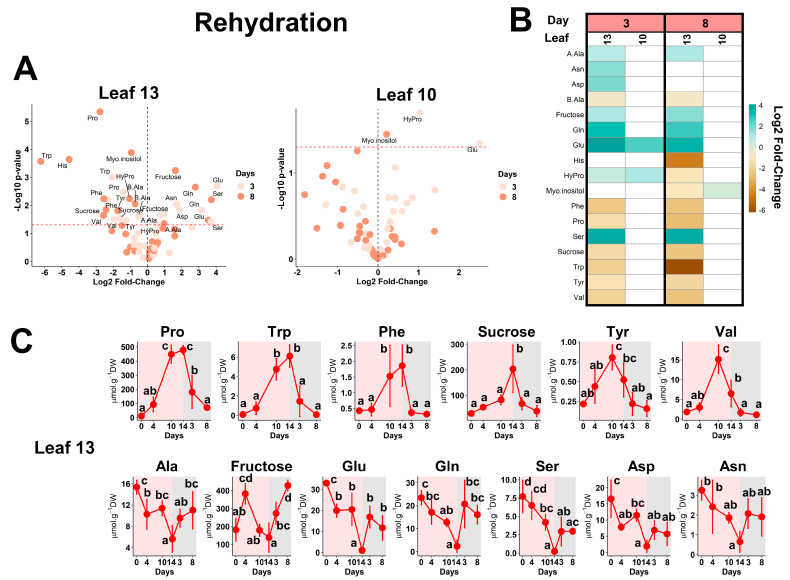
**Metabolic markers of WOSR dehydration/rehydration.** (**A**) Volcano plots. The red line indicates the confidence limit (*p*-value < 0.05). Metabolite names are indicated for the most contrasting and significant fold-changes. (**B**) Heatmap for significant fold-changes. White boxes corresponded to non-significant fold-changes. For (**A**,**B**), analyses were performed by comparing the metabolite contents in rehydrated plants (after 3 and 8 days) versus those detected after 14 days of water stress (time point 0 of rehydration). (**C**) Absolute quantifications for selected contrasted metabolites in leaf 13 during water stress (0 to 14 days, pink panel) and rehydration phases (3 and 8 days, grey panel). Values represent the mean ± SD for 3–5 biological replicates. Statistical comparisons for the different time points were achieved with an ANOVA followed by a post hoc Tukey HSD test with a *p*-value < 0.05. The complete dataset is available in Table S1.

Following water deprivation, the metabolic plasticity of WOSR was evaluated after 3 and 8 days of rehydration in high- and low- N inputs. In this analysis, we only considered leaves 13 and 10 because leaf 7 had fallen during drought for W-stress plants and before 3 days of rehydration for NW-stress plants. Fold-change comparisons were performed with respect to the day 14 for W-stress and NW-stress plants (=day 0 of the rehydration kinetic) (Figure 1A). Overall, the rehydration of W-stress plants only impacted the metabolic profile of leaf 13 (sink leaf), with significant variations for only 17 metabolites (Figure 5A,B). These variations were relatively important (log2 fold-change values ranging between −6 and 4) and comprised both the accumulation and depletion of multiple amino acids and sugars/polyols. Interestingly, most of these metabolite variations showed opposite trends between the drought period and the rehydration phase (Figure 5C). They there-therefore represented relevant metabolic markers for the water status of the younger/developing leaf tissues. Notably, Pro, Phe, Trp and Val contents were increased during drought and were decreased during rehydration. We noted remarkable metabolic plasticity with a return, after only 3 days of rehydration, to the basal state observed in control plants (day 0).

Flexible adjustments were also observed for other amino acids (Ala, Gln, Glu): a significant decrease during drought followed by a significant accumulation during rehydration. Overall, all these metabolites identified as important metabolic markers for dehydration/rehydration of WOSR sink leaves were possibly direct actors for C and N remobilization in directions essential to the acclimation of plants to fluctuating water conditions.

**Figure 6:**
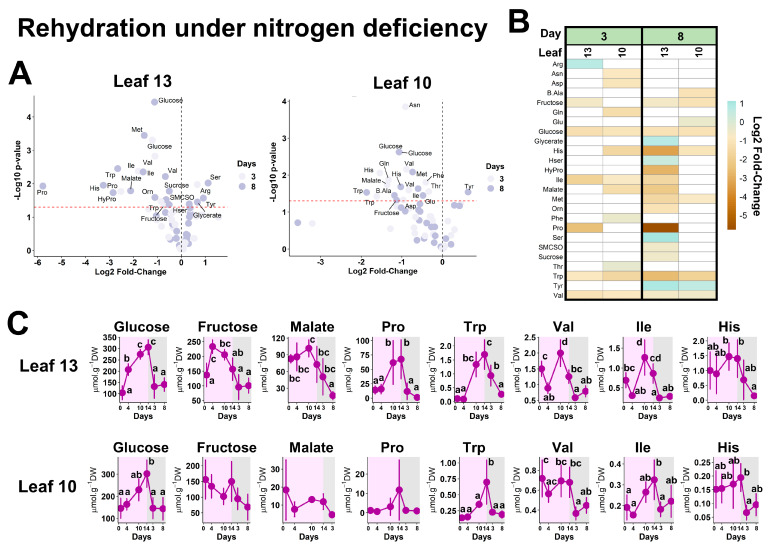
**Metabolic markers of WOSR dehydration/rehydration under N deficiency.** (**A**) Volcano plots. The red line indicates the confidence limit (*p*-value < 0.05). Metabolite names are indicated for the most contrasting and significant fold-changes. (**B**) Heatmap for significant fold-changes. White boxes corresponded to non-significant fold-changes. For (**A**,**B**), analyses were performed by comparing the metabolite contents in rehydrated plants (after 3 and 8 days) versus those detected after 14 days of water stress (time point 0 of rehydration). Analyses were performed only in leaves 10 and 13. Leaf 7 had fallen before day 3 of the rehydration. (**C**) Absolute quantifications for selected contrasted metabolites in the NW-stress condition (0 to 14 days, purple panel) and rehydration (days 3 and 8, grey panel). Values represent the mean ± SD for 3–5 biological replicates. Statistical comparisons for the different time points were achieved with an ANOVA followed by a post hoc Tukey HSD test with a *p*-value < 0.05. The complete dataset is available in Table S1.

For NW-stress plants, the rehydration essentially reflected a decrease in some amino acids and sugars (Figure 6A,B). Regarding leaf 10, only glucose and Trp showed significant variations according to the water status (Figure 6C). Pro, Phe, Trp and Val were also found among the most relevant metabolites in leaf 13. However, glucose, fructose and malate seemed to represent specific metabolic markers of WOSR dehydration/rehydration for N-deficient plants. Nevertheless, only sink leaves of N-deficient plants were subjected to metabolic plasticity during both dehydration and rehydration (Figure 1D). Overall, our analysis identified Pro, Phe, Trp and Val as preponderant metabolic markers of WOSR water status in both high- and low-N conditions, while glucose and fructose represented additional metabolic markers specifically devoted to low-N conditions.

## 3. Discussion

Climate change and the socio-economical context will increase the occurrence of episodes of drought and low-N conditions over the coming years in Europe [8,42]. However, low-N conditions can severely impact amino-acid metabolism functioning. Given its implication in key metabolic functions solicited during acclimation to drought (source-to-sink remobilization, proline biosynthesis, mitochondrial activity), low N could significantly modify the metabolic response of sink and source leaves to drought [19,24,43]. Here, we investigated the effect of low-N conditions on the metabolic response of sink and source leaves of WOSR during drought and rehydration phases. The study was focused on the main polar metabolites containing C and N atoms, as they are fundamental attributes of central metabolism sustaining nutrient assimilation, resource recycling and abiotic stress-induced adjustments.

**Figure 7:**
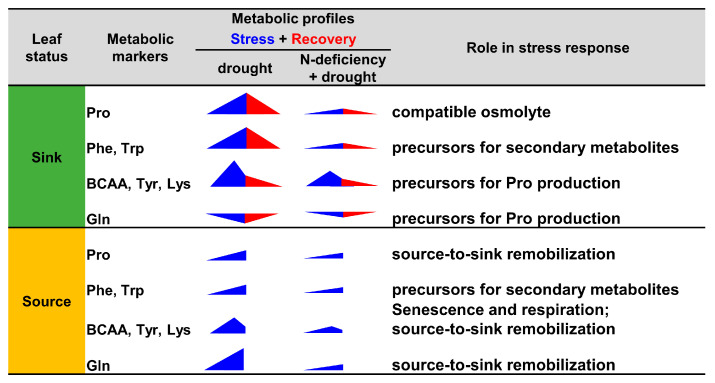
Schematic summary of amino acid variations in sink and source leaves of oilseed rape during drought stress and rehydration under high- and low-N conditions.

Among the metabolic markers identified for drought/rehydration phases, the well-known compatible osmolyte Pro was observed in both high- and low-N conditions (Figure 7). This result clearly reflected the concerted regulation of Pro biosynthesis/degradation that has been already described in WOSR under osmotic and water stress conditions [40,44,45,46]. However, Pro accumulation was drastically reduced in N-deficient plants compared to control plants. Here, WOSR response to N deficiency induced a reduction of plant growth and a decrease in the levels of major amino acids (Ala, Asp, GABA, Glu, Gln, SMCSO) (Figure 2C, [40]). Using the Darmor genotype of WOSR, an important decrease in Ala, Asp, Asn, Glu and Gln in the sink and source leaves was also observed after 56 days of growth in low-nitrate conditions [37]. Thus, N deficiency could essentially decrease WOSR capacity to accumulate amino acids during drought, thereby explaining the quantitative differences of amino acid level modifications observed between NW-stress and W-stress plants (Figure 3 and Figure 4). However, previous results showed that: (i) NW-stress plants were less dehydrated compared to W-stress (Figure 1C), (ii) maximal capacity for proline production remained relatively important in N-stress and NW-stress plants [40]. Therefore, the low levels of Pro found in NW-stress plants compared to W-stress plants mostly reflected a reduction of stress perception rather than a reduction of metabolic response capacity.

Here, we observed an accumulation of BCAAs (Val, Leu, Ile) and other respiratory amino acids (Lys, Tyr) in the sink and source leaves of W-stress and NW-stress plants (Figure 7). This metabolic signature may be explained by different but complementary physiological mechanisms. Accumulation of BCAA in source leaves is usually observed during carbon-starving conditions (dark-induced senescence or prolonged periods of drought) [22]. Such conditions could occur for leaf 7 based on the evolution of major carbohydrates/organic acids and the senescence pattern observed from the same experimental setup [40,47]. Since amino acid biosynthesis is reduced during senescence in source leaves of WOSR, accumulation of BCAA, Lys and Tyr likely originated from senescence-induced protein degradation [48]. However, the maintenance of these amino acid levels in source leaves could also question their utilization by mitochondrial respiration or their potential source-to-sink transport. The source-to-sink transport of BCAAs has not been formally identified in oilseed rape although some amino acid permease transporters with good substrate specificity for BCAAs are positively regulated by drought in plants [49,50,51]. However, the weak enrichment of leaf phloem sap in BCAAs compared to major amino acids (Gln, Glu, Asp, Asn) in different plant species suggests a weak contribution to global N remobilization [52,53,54]. Indeed, Gln, a major amino acid transported from source to sink leaves [19,55], accumulated in much larger amounts (Figure 7). Regarding the young leaf 13 of drought-stressed WOSR, the accumulation of BCAAs could be linked to either the source-to-sink transport of BCAAs, a growth arrest, or Pro biosynthesis (Figure 7). In *Arabidopsis*, drought-induced growth arrest led to the accumulation of many plant metabolites [56]. However, the transient accumulation of BCAAs in W-stress plants suggests other mechanisms, perhaps linked to Pro biosynthesis. A recent study in *Arabidopsis* suggested that amino acids arising from protein degradation were all involved in Pro production during drought [57]. Thus, transient BCAA accumulation could reflect both concomitant production (source-to-sink transport, protein degradation) and consumption events (Pro biosynthesis). Nevertheless, the drought-dependent accumulation of proteases inhibitors in oilseed rape can question whether protein degradation occurred in sink leaves [58,59]. Perhaps additional sources of carbon are involved. A recent study proposed the contribution of starch degradation to proline biosynthesis under long-term osmotic stress in *Arabidopsis* [60]. Additional ^13^C-labelling experiments in *B. napus* will help to identify the respective contributions of soluble proteins degradation, photosynthesis/starch, and carbon imports from source leaves to proline production in sink leaves [61,62,63].

Our results also identified the aromatic amino acids Phe and Trp as valuable metabolic markers in WOSR sink leaves for drought/rehydration phases under high- and low-N conditions (Figure 5 and Figure 6). Phe and Trp are synthesized from the shikimate pathway and serve as precursors for several metabolite classes: (i) phenylpropanoids, flavonoids, phenylalkyl glucosinolates from Phe, and (ii) indoles glucosinolates, indoles alkaloids, salicylates, auxins from Trp [64]. Previous studies in different crops showed that drought alone significantly increased the amount of these amino acids and stimulated several metabolic pathways associated with phenylpropanoids, flavonoids and phytohormones [22,30,39,65]. These secondary metabolite classes are closely correlated with plant capacity to tolerate drought, perhaps because they can mitigate oxidative stress and regulate plant growth [27,29,66]. Thus, the accumulation of Phe and Trp in WOSR during drought could be linked to possible secondary metabolite biosynthesis (Figure 7). Indeed, the maintenance of glucosinolates levels, a class of specialized metabolites specifically found in *Brassica* species, is essential for drought tolerance [67,68,69]. In the genotype Tenor used in this study, 43% of the total leaf glucosinolate content was derived from Phe and Trp (phenylalkyl and indolic glucosinolates) [70]. Clearly, the variation of Phe and Trp levels in WOSR leaves during acclimation to drought/rehydration was likely associated with the activity of specialized metabolism.

Finally, sugar metabolism showed an interesting metabolic reconfiguration in sink leaves during drought. Notably, sucrose was accumulated in leaf 13 under both W-stress and NW-stress conditions (Figure 3D and Figure 4C). This accumulation could be linked either to drought-induced stomatal closure or to osmotic adjustment [25,71,72]. However, the total content of sucrose, glucose and fructose remained weakly changed in drought-stressed plants proposing this osmo-regulating role (Figure S5). In addition, leaf 9 from the same experimental setup maintained stomatal closure after rehydration [40], while a decrease in sucrose was observed during rehydration for leaf 13 (Figure 5). The decrease in plant growth and the activation of starch catabolism or source-to-sink transports by drought are also additional factors to consider [56,73,74]. Given the important decrease in sucrose content in the source leaf 7 of both W-stress and NW-stress conditions, the source-to-sink transport of sucrose should be carefully investigated in subsequent studies dealing with drought and N deficiency.

## 4. Materials and Methods

### 4.1. Plant Growth, Stress Application and Leaf Harvest

The WOSR leaves analyzed in this study were obtained from a previous experiment [40]. Briefly, three-week-old seedlings of the *Brassica napus* genotype Tenor, grown under greenhouse conditions in 2 L pots filled with perlite, were irrigated for two weeks with a nutritive solution containing 8 mM nitrate as the sole N source (“control”). Then, N deficiency was triggered for half of the plants by reducing the availability of the N source to 0.4 mM nitrate (“N-stress”). After two weeks, plants for each N regime (control or N-stress) were subjected or not to drought stress by completely stopping irrigation for 14 days (“W-stress” and “NW-stress”). Rehydration of the water-stressed plants was performed for up to 8 days with the nutritive solutions containing 8 or 0.4 mM of nitrate. For the well-watered plants, irrigation was maintained with 8 mM (“control”) or 0.4 mM nitrate (“N-stress”) regimes throughout the culture (Figure 1A). Five plants per treatment were sampled. Leaf ranks were identified and numbered based on the date of ontogenetic appearance from the oldest to youngest (bottom to top of the plant). The experimental setup was previously validated by quantification of chlorophyll content and relative water content in the leaves for each condition (“control”, “N-stress”, “W-stress”, “NW-stress”) on days 0, 4, 10, 14, 17 (3 days of rehydration for drought plants) and 22 (8 days of rehydration for drought plants). In the present study, we selected leaves 13, 10 and 7 for metabolic analysis, representing a total of 288 samples.

### 4.2. Polar Metabolite Extraction

Frozen leaves were ground into a fine powder and after lyophilization 10 mg of dry weight was incubated with 400 µL MeOH containing two internal standards (200 µM DL-3-aminobutyric acid (BABA) and 400 µM adonitol). After 15 min of incubation at room temperature with orbital shaking, 200 µL of CH_3_Cl was added to the mixture. After a second incubation step (same conditions as before), 400 µL of ultrapure H_2_O was added. The samples were vigorously mixed for 10 s and centrifuged at 12,000× *g* at room temperature for 5 min. The upper phase (MeOH/H_2_O fraction) containing polar metabolites was recovered. For each sample, two aliquots of 50 µL were evaporated using a SpeedVac concentrator at 35 °C for 4 h for subsequent metabolite separation and quantification.

### 4.3. Quantification of Amino Acids

Amino acids from dried aliquots were derivatized according to the AccQTag Ultra Derivatization Kit protocol (Waters). Then, 1 µL of derivatized amino acids was injected into an Acquity UPLC system (Waters) with an Acquity UPLC^®^ BEH C18 1.7 μm × 2.1 mm × 100 mm column heated at 55 °C. Elution was performed following a specific gradient, described in [75]. Derivatized amino acids were detected with a photodiode array detector set at 260 nm. Peak identity was confirmed according to the elution time from a mixture of commercial amino acids (Figure S1). BABA was used as an internal standard since it did not interfere with chromatographic elution of targeted amino acids. The purity of the internal standard peak was previously confirmed by mass spectrometry using the same column/elution procedure with a UPLC-DAD-TQD system [46]. Peak integration was performed with Empower 2 software (Waters) and was visually inspected to correct potential errors of identification/integration. Peak areas below a signal-to-noise ratio of 4 were considered as not detected (missing values). Raw data were normalized to the area of the internal standard (BABA), the response factor of commercial amino acids compared to the internal standard (using an external calibration curve), and the sample dry weight. Amino acid contents were expressed in µmol·g^−1^ DW. Total amino acid contents were quantified by summing the amino acid contents detected in the analysis.

### 4.4. Quantification of Sugars, Polyols and Organic Acids

Sugars, polyols and organic acids from dried aliquots were derivatized using methoxyamine (MEOX) and N-methyl-N-trimethylsilyl trifluoroacetamide (MSTFA). Briefly, dried aliquots were resuspended in 50 µL of freshly prepared MEOX-HCl dissolved in pyridine (20 g/L) and incubated for 90 min at 30°C with orbital shaking. Then, 50 µL of MSTFA was added, and another incubation step was performed for 30 min at 37 °C with orbital shaking. Subsequently, 1 µL of derivatized metabolites was injected into a gas chromatography-flame ionization detector system (GC-FID) Trace 1300 (Thermo-Fisher Scientific, Waltham, MA, USA) with a TG 5MS 30 m × 0.32 mm × 0.25 mm column using the following parameters: split mode 1/20, injector at 260 °C, and FID at 310 °C. Elution was performed following a specific gradient, described in [76]. Peak identity was confirmed according to the elution time from a mixture of commercial standards (Figure S2). Adonitol was used as an internal standard since it did not interfere with the chromatographic elution of targeted compounds. The purity of the internal standard peak was previously confirmed by mass spectrometry using the same column/elution procedure with the GC-MS system [61]. Peak integration was performed with Chromeleon software (Thermo-Fisher Scientific) and was visually inspected to correct potential errors of identification/integration. Peak areas below a signal-to-noise ratio of 4 were considered as not detected (missing values). Raw data were normalized to the area of the internal standard (adonitol), the response factor of commercial amino acids compared to the internal standard (using an external calibration curve), and the sample dry weight. Sugars, polyols and organic acids contents were expressed in µmol·g^−1^ DW. Total sugar/organic acid contents were quantified by summing the sugar/organic acid contents detected in the analysis.

### 4.5. Data Management, Visualization and Statistical Analyses

Raw data were retreated and visualized using custom R scripts based on the packages tidyr, dplyr and ggplot2 and built-in functions from RStudio v2022.07.2 build 576 [77]. Principal component analysis (PCA) was carried out with the package mixOmics. In this analysis, we used the half-minimum method for missing value imputation [78]. For volcano plots, log2 fold-changes were calculated as the log2 of the ratio between the mean of two groups of values (a filter was applied to only consider groups with a minimum of three values). Accordingly, *p*-values were calculated using a Student’s *t*-test (two-sided, unequal variance) for comparison of two groups of values (a filter was applied to only consider groups with a minimum of three values). Heatmap representation was achieved with the package Pheatmap. Subsequent comparisons of two groups of values were carried out with a Student’s *t*-test (two-sided, unequal variance) using built-in functions from RStudio. Comparison of multiple groups of values was achieved with an ANOVA followed by a post hoc Tukey HSD test using the package Multcomp.

## 5. Conclusions

This study provided a general overview of the acclimation of central metabolism in sink and source leaves of oilseed rape under a combination of water and N-limiting conditions. Besides the well-known metabolic marker Pro, we identified Phe and Tyr as important metabolic indicators of oilseed rape water status in high- and low-N conditions. Given their role as metabolic precursors, these results suggested a contribution of central metabolism in the *de novo* production of specialized metabolites during abiotic stresses. BCAA, Lys and Tyr were transiently accumulated during drought in the sink and source leaves. This specific pattern likely reflected different physiological mechanisms: stress-induced senescence in source leaves, source-to-sink remobilization of BCAAs, proline production in sink leaves, and an arrest of growth. Overall, N deficiency essentially reduced the level of major amino acids and subsequently the quantitative modulation of leaf central metabolite levels in response to drought. However, this quantitative effect mostly reflected a reduction of stress perception rather than a reduction of metabolic response capacity. Hence, the metabolic acclimation of plants to a combination of abiotic stresses cannot be necessarily deduced from the effects of each abiotic stress taken separately. In the future, the metabolic markers of water status identified here in high- and low-N conditions may highlight new quantitative traits of interest for QTL research in oilseed-rape breeding programs for improving N use efficiency under water restriction.

## Data Availability

The original contributions presented in the study are included in the article/Supplementary Materials, and further inquiries can be directed to the corresponding authors.

## Supplementary Materials

The following supporting information can be downloaded at: https://www.mdpi.com/article/10.3390/plants13070969/s1, Figure S1: Chromatographic separation of amino acids by UPLC-DAD; Figure S2: Chromatographic separation of sugars, polyols, and organic acids by GC-FID; Figure S3: Global principal component analysis; Figure S4: Leaf amino acid contents under nitrogen deficiency; Figure S5: Total sugars and organic acid contents during 14 days of drought; Table S1: Complete metabolic dataset generated by this study.
