# Supplementary material for: Low Nitrogen Input Mitigates Quantitative but Not Qualitative Reconfiguration of Leaf Primary Metabolism in Brassica napus L. Subjected to Drought and Rehydration
Source: Plants (Basel). 2024 Mar 27;13(7):969. doi: 10.3390/plants13070969 (PMC11013775; doi:10.3390/plants13070969)
Supplement: Supplementary file 1 [file plants-13-00969-s001.zip › Supplemental figures.pdf]

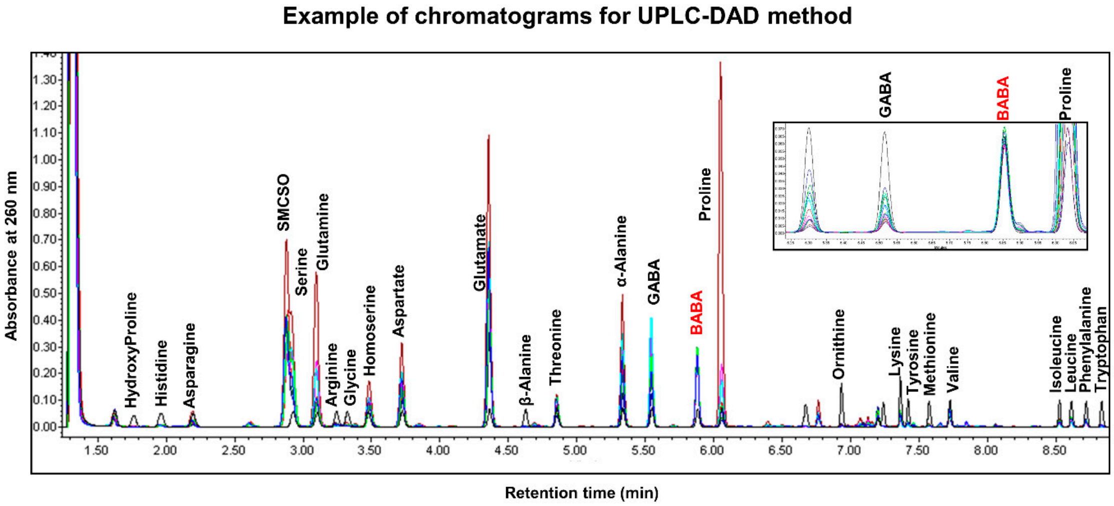

Figure S1. Chromatographic separation of amino acids by UPLC-DAD. The internal standard (BABA) is shown in red, with a detailed zoom of the elution window in the inset. The purity of the internal standard peak was previously confirmed by mass spectrometry using the same column/elution procedure with an UPLC-DAD-TQD system.

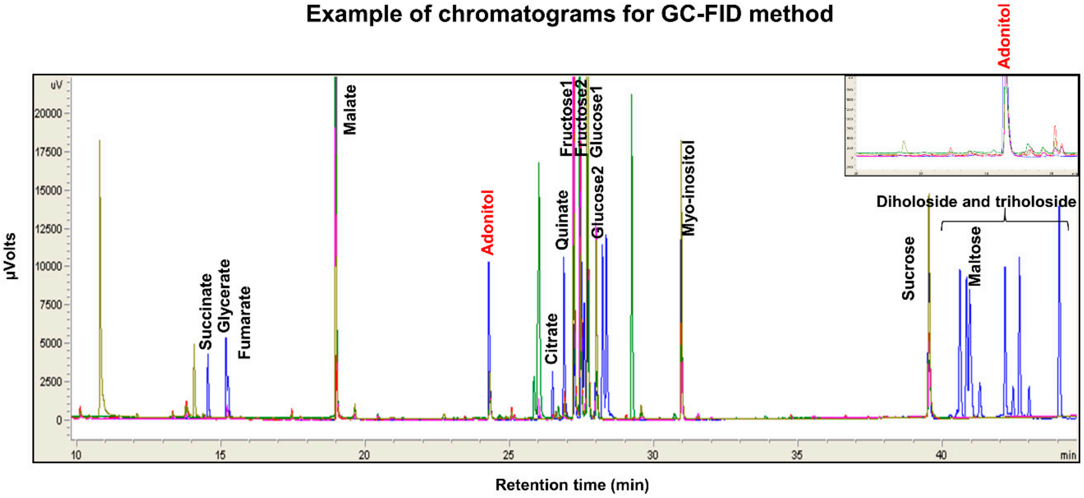

Figure S2. Chromatographic separation of sugars, polyols and organic acids by GC-FID. The internal standard (Adonitol) is shown in red, with a detailed zoom of the elution window in the inset. The purity of the internal standard peak was previously confirmed by mass spectrometry using the same column/elution procedure with an GC-MS system.

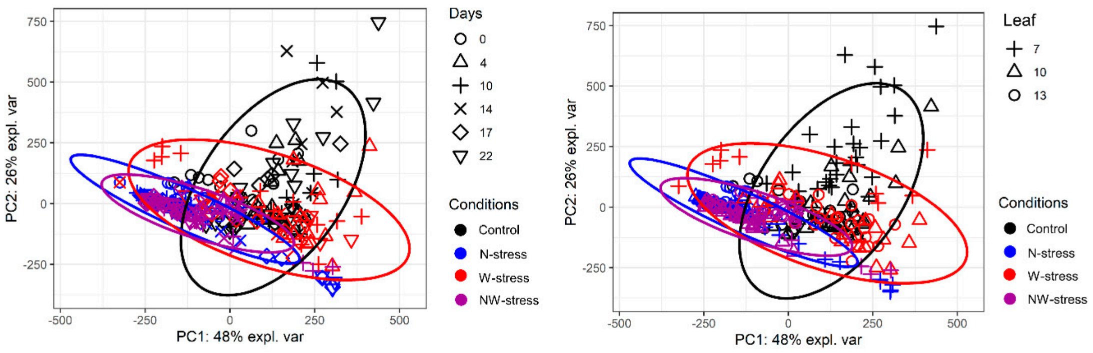

Figure S3. Global principal component analysis. Principal component analysis of metabolite profiling data for the leaves 7, 10 and 13 in oilseed rape plants (control, N-, W- or NW- stress) during the 22 days of experiment. PCA was performed with a total of 36 metabolites, with 3-5 biological replicates per condition, time-point and leaf.

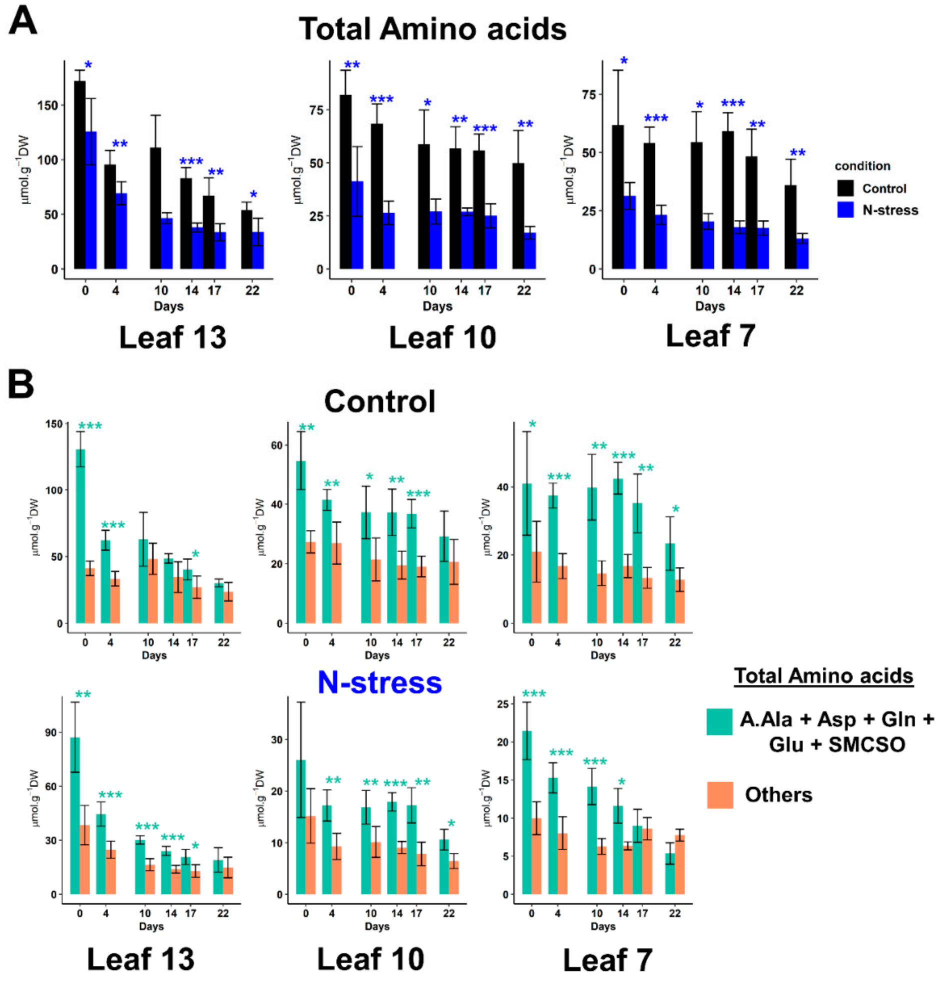

Figure S4. Leaf amino acid contents under nitrogen deficiency. (**A**) Total amino acid content and (**B**) contribution of major amino acids. Values represent the mean ± SD for 3-5 biological replicates. Statistical differences between control and N-stress conditions for each kinetic point are denoted with stars according to a Student's t-test (*, p-value<0.05; **, p-value<0.01; ***, p-value<0.001). The complete dataset in available in **Table S1**.

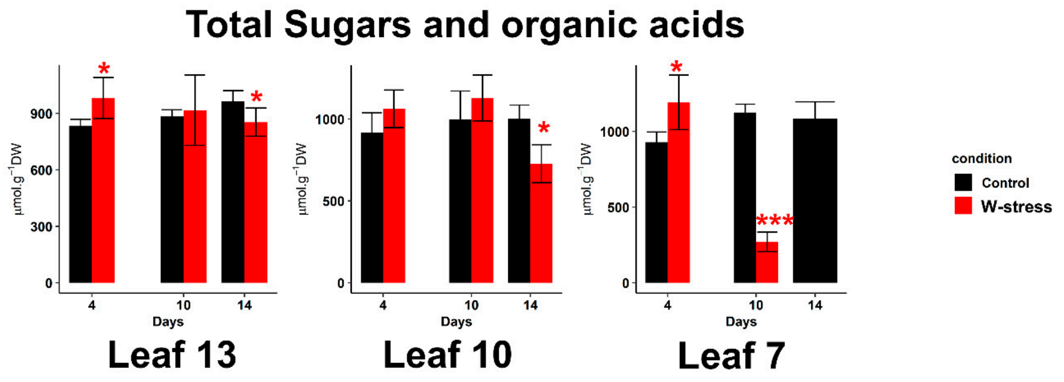

Figure S5. Total sugars and organic acid contents during 14 days of drought. Values represent the mean ± SD for 3-5 biological replicates. Statistical differences between control and W-stress conditions for each kinetic point are denoted with stars according to a Student's t-test (*, p-value<0.05; **, p-value<0.01; ***, p-value<0.001). The complete dataset in available in **Table S1**.
